# The complete chloroplast genome of *Swertia cordata*

**DOI:** 10.1080/23802359.2019.1681310

**Published:** 2019-10-30

**Authors:** Li Huang, Chunlin Chen, Qiao Yang, Wenjie Yang, Quanjun Hu

**Affiliations:** Key Laboratory of Bio-Resource and Eco-Environment of Ministry of Education, College of Life Sciences, Sichuan University, Chengdu, Sichuan, P. R. China

**Keywords:** *Swertia cordata*, chloroplast genome, phylogenetic analysis

## Abstract

*Swertia cordata*, a herbaceous plant, has been used in medicines as an alternative febrifuge and anthelmintic. In this study, the complete chloroplast DNA sequence of *S. cordata* was assembled. It is 153,429 bp in length, including a large single-copy region of 83,613 bp and a small single-copy region of 18,038 bp separated by a pair of inverted repeat regions of 25,889 bp each. A total of 129 genes are detected, including 84 protein-coding genes, 37 tRNA genes, and 8 rRNA genes. The complete chloroplast genome of *S. cordata* will help to study the genetic diversity and phylogenetic analysis of *Swertia*.

Swertia *cordata*, one of the important medicinal plant species distributed between 2400–3600 m a.s.l., is an annual, erect, solitary or tufted branched herb with yellowish-white flowers. The plant has been used in medicines as an alternative febrifuge and antihelmintic and as a bitter tonic (Khan and Haqqani 1981). In this study, we reported the complete chloroplast genome of *S. cordata* based on the next-generation sequencing data and uncovered its phylogenetic relationship with eight Gentianaceae species and one Apocynaceae species of which complete chloroplast genomes are available.

We collected its fresh leaves from a single individual from Tengchong county in Baoshan, Yunnan, China (N 25°26′, E 98°44′) and dried them with silica gels. A voucher specimen (RC56) of *S. cordata* was deposited in the Herbarium of Sichuan University, Chengdu, China. We isolated the total genomic DNA from fresh leaves using a modified hexadecyltrimethylammonium bromide (CTAB) method (Doyle and Doyle 1987) and sequenced it based on the Illumina pair-end technology. Around 6 Gb clean reads were assembled against the complete chloroplast genome of *Swertia mussotii* (GenBank accession no. NC_031155.1) using the programme NOVO-Plasty (Dierckxsens et al. 2017). The annotations of chloroplast genome were conducted by the programme Plann (Huang and Cronk 2015), and we corrected the annotations manually with Geneious (Kearse et al. 2012). The annotated complete chloroplast DNA of *S. cordata* has been deposited into GenBank with the accession number of MK955906.

The size of the complete chloroplast genome of *S. cordata* is 153,429 bp. It is a circular DNA and contains two copies of inverted repeats (IRs) (IRa and IRb, 25,889 bp each) separating the large single-copy region (LSC) of 83,613 bp and the small single-copy region (SSC) of 18,038 bp. In addition, we annotated a total of 129 genes including 84 protein-coding genes, 37 tRNA genes, and 8 rRNA genes. Most genes occur as a single copy, however, 4 rRNA genes (i.e. 4.5S, 5S, 16S, and 23S rRNA), 7 protein-coding genes (i.e. *rps12*, *ycf2*, *ycf15*, *rpl23*, *rpl2*, *rps7*, and *ndhB*), and 7 tRNA genes (i.e., *trnL-CAA*, *trnV-GAC*, *trnI-GAU*, *trnA-UGC*, *trnR-ACG*, *trnN-GUU*, and *trnI-CAU*) occur in duplicated copies. The GC-content of the complete chloroplast genome is estimated to be 38.1%.

We used RAxML (Stamatakis 2014) with 1000 bootstraps under the GTRGAMMAI substitution model to reconstruct a maximum likelihood (ML) phylogeny of eight published complete chloroplast genomes of Gentianaceae, using *Nerium oleander* (Apocynaceae) as an outgroup. The result of the phylogenetic analysis indicates that *S. cordata* is closed to *Swertia verticillifolia* and *Swertia mussotii* (Figure 1). The complete chloroplast genome of *S. cordata* will help to study the genetic diversity and phylogenetic analysis of *Swertia*.

**Figure 1.**
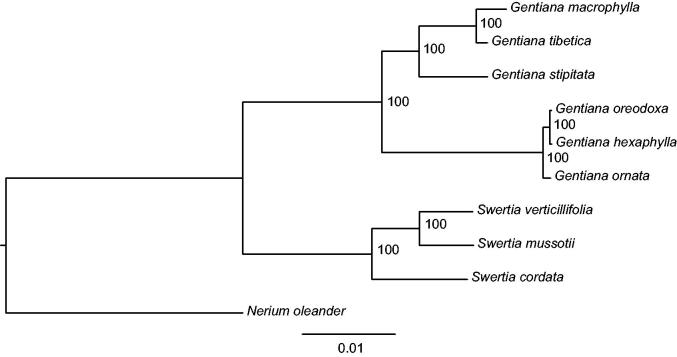
Phylogenetic relationship of the *S. cordata* chloroplast genome with nine previously reported complete chloroplast genomes. Bootstrap percentages are indicated for each branch. GenBank accession numbers: *Swertia verticillifolia* (MF795137.1), *Swertia mussotii* (NC_031155.1), *Gentiana tibetica* (NC_030319.1), *Gentiana macrophylla* (NC_035719.1), *Gentiana oreodoxa* (NC_037982.1), *Gentiana hexaphylla* (NC_037980.1), *Gentiana stipitata* (NC_037984.1), *Gentiana ornata* (NC_037983.1), *Nerium oleander* (NC_025656.1).
